# Exogenous Ketone Salt Supplementation and Whole-Body Cooling Do Not Improve Short-Term Physical Performance

**DOI:** 10.3389/fnut.2021.663206

**Published:** 2021-07-15

**Authors:** Daniel Clark, Stephanie Munten, Karl-Heinz Herzig, Dominique D. Gagnon

**Affiliations:** ^1^Laboratory of Environmental Exercise Physiology, School of Kinesiology and Health Sciences, Laurentian University, Sudbury, ON, Canada; ^2^Southwest College of Naturopathic Medicine and Health Sciences, Tempe, AZ, United States; ^3^Centre for Research in Occupational Safety and Health, Laurentian University, Sudbury, ON, Canada; ^4^Research Unit of Biomedicine, Medical Research Center, University of Oulu, Oulu, Finland; ^5^Institute of Pediatrics, Poznan University of Medical Sciences, Poznan, Poland; ^6^Department of Sports and Exercise Medicine, Clinicum, Faculty of Medicine, University of Helsinki, Helsinki, Finland

**Keywords:** exogenous ketone salts, ergogenic aids, cooling, exercise metabolism, performance

## Abstract

Exogenous ketone supplementation and whole-body cooling (WBC) have shown to independently influence exercise metabolism. Whether readily available ketone salts, with and without WBC, would provide similar metabolic benefits during steady-state aerobic and time-trial performances was investigated. Nine active males (VO_2peak_: 56.3 ± 2.2 mL·kg^−1^·min^−1^) completed three single-blind exercise sessions preceded by: (1) ingestion of placebo (CON), (2) ketone supplementation (0.3 g·kg^−1^ β-OHB) (KET), and (3) ketone supplementation with WBC (KETCO). Participants cycled in steady-state (SS, 60% *W*_max_) condition for 30-min, immediately followed by a 15-min time trial (TT). Skin and core temperature, cardio-metabolic, and respiratory measures were collected continuously, whereas venous blood samples were collected before and after supplementation, after SS and TT. Venous β-OHB was elevated, while blood glucose was lower, with supplementation vs. CON (*p* < 0.05). TT power output was not different between conditions (*p* = 0.112, CON: 190 ± 43.5 W, KET: 185 ± 40.4 W, KETCO: 211 ± 50.7 W). RER was higher during KETCO (0.97 ± 0.09) compared to both CON (0.88 ± 0.04, *p* = 0.012) and KET (0.88 ± 0.05, *p* = 0.014). Ketone salt supplementation and WBC prior to short-term exercise sufficiently increase blood β-OHB concentrations, but do not benefit metabolic shifts in fuel utilization or improve time trial performance.

## Introduction

Nutritional fueling strategies (i.e., carbohydrate and protein loading, sport drinks, stimulants, and creatine) and attenuation of the rise in core body temperature (i.e., cold water immersion, or wearing a cooling vest during a race) are commonly used to influence metabolism and improve human performance during both short and prolonged exercises (1, 2). The use of exogenous ketone supplementation (acetoacetate, β-hydroxybutryrate; β-OHB) has gained popularity as a nutritional strategy to increase athletic performances due to its ability to influence skeletal muscle metabolism and act as a signaling metabolite (3–5). Metabolically, ketone supplementation has been proposed to maintain carbohydrate (CHO) sources by providing an alternative fuel source, decreasing CHO oxidation, coupled with a concurrent increase in fat utilization (5). As such, reserved CHO sources could be utilized during short, high-intensity exercise, such as an end-spurt during a race. Whole-body cooling strategies reduce performance impairments associated with excessive increases in core temperature (1), but also have an effect on metabolism by lowering glycogenolytic rates during exercise and increasing oxidative metabolism *via* greater fat oxidation (6–8). Interestingly, it is unknown how combining these two strategies would influence substrate partitioning, and subsequently, exercise performance.

Ketone bodies are generated endogenously in a state of CHO restriction or insulin deficiency. Elevated circulating ketone bodies increases the concentration of acetyl-CoA and NADH+, from mitochondrial oxidative phosphorylation of ketones, and reduces in glycolytic intermediates, mainly pyruvate, without influencing TCA metabolites (3, 9). Consequently, exogenous ketone supplementation should theoretically result in an increased reliance on ketone and intramuscular fat oxidation (3). However, this finding is equivocal between ketone ester studies (3, 9), and these alterations have yet to be shown with ketone salt supplementation. There is evidence that when ketones are supplemented exogenously *via* ketone monoester [(R)-3-hydroxybutyl (R)-3-hydroxybutyrate (βHB-BD)], blood ketone concentrations can increase to a greater extent than can be produced endogenously (~3 vs. 1 mM) (3, 10, 11), while still allowing CHO substrates to be readily available (5, 12). The influence of ketone supplementation on endurance exercise was first demonstrated by Cox et al. (3). They found that endurance athletes, supplemented with 573 mg·kg^−1^ of ketone monoester, had slight performance improvements (2%) during a self-paced 30-min cycling time trial (TT). Changes in performance were coupled with lower lactate and higher βHB concentrations, suggesting glycogen/glucose preservation during the 60 min of steady-state exercise (75% *W*_max_) prior to the TT (3). O'Malley et al. (13) investigated the effects of these commercially available ketone salts in healthy adult males during short, high intensity cycling exercise. While they observed a significant decrease in the respiratory exchange ratio (RER) during steady state exercise, ketone salt supplementation reduced power output (7%) during a subsequent 150 kJ TT (13). It should be noted that the majority of research on the effects of ketone ester supplementation has focused on prolonged endurance exercise (3, 11), while the effect of ketone salts has been studied over a variety of exercise lengths (14) (long duration exercise (>60 min) (15); short, high-intensity exercise (≤25 min) (16, 17); moderate duration exercise durations (25–50 min) (13, 18, 19).

A rise in core body temperature during exercise has a direct relationship with performance degradation and heat illness (20, 21). An excessive increase in core and muscle temperature has consistently shown to result in greater whole-body CHO oxidation, with an associated increase in muscle and blood lactate accumulation, which may drive an earlier onset of fatigue (22, 23). Strategies have been developed to attenuate rises in core body temperature in order to maximize performance outcomes, including precooling and percooling (cooling during exercise) (24–27). Additionally, whole-body cooling is well-known to regulate the metabolism of lipids and CHO differently than a thermoneutral environment. While shivering thermogenesis at rest in the cold elicits greater CHO metabolism, the metabolism of fats is favored during exercise (7, 8, 28). Cold exposure increases sympathetic nervous system activity and the secretion of catecholamines, increasing whole-body lipolysis (28, 29). Cold exposure does not influence ketone concentrations (7), therefore, the combination of increased plasma ketone concentrations and whole-body cooling could result in altered steady-state exercise metabolism, sparing CHO. However, whether the combination of ketone salt supplementation and cooling would further alter metabolism and physical performance is unknown.

The purpose of this study was to investigate the independent effect of exogenous ketone salts, as well as the potential synergistic effects of ketone salt supplementation combined with whole-body cooling, on short-term aerobic exercise substrate metabolism, and high intensity time trial exercise performance in recreationally trained individuals. We hypothesized that ingestion of exogenous ketone salts would lower exercise RER, during submaximal steady-state exercise by contributing to energy utilization, resulting in improved exercise performance during a time trial exercise. Additionally, the combination of ketone salts with cooling may further shift substrate metabolism toward fat oxidation (lower RER) and improve performance.

## Materials and Methods

### Participants

Nine healthy active males were recruited from a university population (age: 21.9 ± 1.7 years, weight: 74.9 ± 9.3 kg, height: 182.8 ± 12.6 cm, %BF: 13.2 ± 2.6%, VO_2peak_: 56.3 ± 2.2 mL·kg^−1^·min^−1^). None of the participants were trained endurance athletes, although inclusion criteria required individuals to be aerobically fit, and we required to have a peak oxygen consumption (VO_2peak_) value of 50.0 mL·kg^−1^·min^−1^ or greater in order to participate in this study. Participants were screened with a Get Active Questionnaire and a health screening form for health conditions or diseases that could be aggravated by cold or exercise. None of the participants were on prescribed medications or following a ketogenic diet. Participants also avoided alcohol consumption, strenuous exercise, caffeine, and tobacco 24 h prior to each experimental exercise session. To control for dietary influences on metabolism during exercise, participants completed a self-reported diet diary the day prior to their first experimental exercise session. Participants were then required to replicate and maintain the same diet the day prior to each remaining exercise session, with the built-in assumption that this energy standardization method yields significant variability in food intake between participants (30). Adherence to diet replication was verbally confirmed prior to beginning each experimental session. In addition, we confirmed that the participants had a baseline blood β-OHB <0.5 mmol·L^−1^, which is typically the lower limit of nutritional ketosis (31). Written informed consent was provided prior to testing. All procedures were in accordance with the Declaration of Helsinki and approved by the University's Research Ethics Board (REB# 6013624).

### Procedures

Participants reported to the laboratory for four sessions: one baseline session and three experimental exercise sessions following a single-blind, counterbalanced design of the following conditions: placebo control (CON), ketone salt supplementation (KET), and ketone salt supplementation with whole-body cooling (KETCO). This study did not include a cooling only group, as the current literature supports that whole-body cooling alone improves performance (20, 24–26), and the experimental design compares the effect of ketone salts with and without whole-body cooling only. Participants completed the experimental sessions at the same time between 07:00 and 10:00 h, following a 10–12 h overnight fast, 7 days apart. During baseline testing, the participant's height, weight and age were obtained upon arrival. Additionally, percent body fat was estimated by the same researcher using skinfold calipers measured at seven sites: tricep, chest, subscapular, midaxillary, suprailiac, abdominal, and thigh (32). Three measurements were obtained from each site, and an average was recorded. Finally, participants completed a ramp protocol on a cycle ergometer to determine VO_2peak_ and maximal power output (*W*_max_). The protocol started at 75 W, increasing by 25 W every 2 min until volitional fatigue. VO_2_ was considered to be peak when at least two of the following criteria occurred, (1) if heart rate did not significantly increase with increasing workload (defined as an increase of no more than 5 bpm), (2) if RER was >1.15, or (3) if VO_2_ did not significantly increase with increasing workload (defined as an increase of no more than 2 ml·kg^−1^·min^−1^). *W*_max_ was determined by the maximum wattage the participant was able to maintain for a full 2 min (i.e., the last fully completed stage of the ramp protocol).

For the experimental testing, participants were instrumented (see “Instrumentation” section below) upon arrival and given the ketone salt or a taste-matched placebo mixed in a 500 ml water solution (see “Supplement” section below). Then, they immediately entered a climate controlled environmental chamber set at either thermoneutral (21°C) or cold (0°C). In the cold condition, participants were also fitted with a cooling vest lined with ice packs (FlexiFreeze, Mequon, WI, USA). Participants were required to wear the same clothes in each condition; a simple T-shirt and shorts. Participants then sat in the chamber for 30 min to allow absorption of ketones into the blood (5, 12).

After 30 min of seated rest, the participant began the exercise protocol. The protocol consisted of 30 min steady-state preload cycling (SS), followed by a 15-min time trial (TT), on a cycling ergometer (CompuTrainer, RacerMate, Seattle, WA, USA). The cycling ergometer was fit with a Trek road bike with the seat height adjustments made for each participant. That height was recorded and reproduced for each trial. During the 30-min SS preload, participants cycled at 60% of *W*_max_. The resistance on the cycling ergometer was automatically adjusted using TrainerRoad software (Denver, CO, USA) to maintain a constant power output. Immediately following the preload, participants began the 15-min TT. The cycle ergometer was set to linear mode and the participants were instructed to cycle as hard as they could for 15 min. No verbal encouragement was provided during the SS or TT. Participants were blinded to all physiological and performance output measures (i.e., cadence, distance, and power output) during the time trial, and were only given verbal elapsed time cues every 2.5 min.

### Instrumentation

Skin temperature was measured *via* wireless surface skin temperature probes (Thermochron iButton, Maxim Integrated, San Jose, CA, USA) at four sites: chest, lateral bicep, lateral thigh, and lateral calf. Core temperature (T_c_) was measures through a rectal thermistor (Physitemp, Clifton, NJ, USA) inserted 10 cm beyond the anal sphincter. Breath-by-breath measurements were measured continuously during exercise using an open-circuit ergospirometer in breath-by-breath mode (K5, Cosmed, Pavona, RM, Italy) to obtain measures of oxygen consumption (VO_2_), carbon dioxide production (VCO_2_), respiratory exchange ratio (RER), and ventilation rate (VE). Gas analyzers were calibrated with air tanks containing 16% O_2_ and 4% CO_2_ and the gas flow sensor was calibrated using a 3 L calibration syringe before each exercise session. Gas flow was measured through a bidirectional pitot tube flow sensor attached to the face-fitting mask worn by all participants during the exercise sessions.

### Supplement

Participants were given a taste and color-matched placebo or the ketone supplement drink 30 min prior to exercise (13, 15, 16). Consumption of the supplement/placebo occurred 30 min prior to testing to achieve maximum blood ketone concentrations during the exercise session (33). The contents of the drink were blind to the participant. The dosing of the racemic ketone salt (50% R-β-OHB salts, 50% S-β-OHB; KetoForce; Prototype Nutrition, Urbana, IL, USA) was given at a dose of 0.3 g·kg^−1^ body mass of β-OHB, 0.01 g·kg^−1^ body mass sodium, and 0.01 g·kg^−1^ body mass potassium, as used in previous studies (13). The placebo also matched the quantities of sodium and potassium in the ketone supplement but provided no other nutrition. This dosage has been shown to produce a blood ketone concentration of ~1 mmol·L^−1^ (13). The success of the blinding of the supplement was not assessed in this study. Participants did not indicate any gastrointestinal issues during the experimental sessions.

### Blood Sampling

Fingertip capillary blood samples were collected using a contact-activated lancet (BD Microtainer, BD, Franklin Lakes, NJ, USA) prior to supplement ingestion (BL), at the end of the pre-exercise condition (PRE; 30 min after supplement ingestion), immediately after the 30-min steady state cycling (SS), and immediately following the 15-min time trial (TT) to assess blood β-OHB and blood glucose (FreeStyle Precision Neo; Abbott Laboratories, Green Oaks, IL, USA). Venous blood samples, *via* a catheter inserted in the antecubital vein, were also collected in plain K2EDTA tubes to assess pH, and concentrations of base excess of extracellular fluid (BEecf), bicarbonate (${\text{HCO}}_{3}^{-}$), partial pressure of oxygen (pO_2_), partial pressure of carbon dioxide (pCO_2_), total carbon dioxide (TCO_2_), lactate, and oxygen saturation (SO_2_) (i-STAT1 Handheld, Abbott Point of Care, Princeton, NJ, USA).

### Data and Statistical Analysis

Mean skin temperature (${\bar{T}}_{\text{sk}}$) was calculated using the following equation (34):

$$\begin{array}{l} \bar{T}sk=0\text{.}3•\left(\text{Tchest }+\text{ Tarm}\right)\;+\;0\text{.}2•\left(\text{Tcalf }+\text{ Tleg}\right) \end{array}$$

Data are reported as mean with standard deviation (mean ± SD). Normality was tested using the Shapiro–Wilk test. A one-way analysis of variance with repeated measures were used to identify differences in mean power output and distance traveled during the time trials, as well as cardiorespiratory measures (RER, VO_2_, and VE) during steady state exercise sessions. Two-way analyses of variance with repeated measures (Factors: condition (levels: CON, KET, KETCO), and time) were used to identify differences in ${\bar{T}}_{\text{sk}}$ and T_c_, blood glucose, blood lactate, blood β-OHB, blood gases. When a significant *F* ratio was observed, pairwise comparisons with Bonferroni correction were conducted. All effects were tested at a 95% confidence interval (*p* ≤ 0.05). To conduct an analysis of power we considered the variability observed in RER from previous studies (13). Assuming the same level of variability, it was estimated that a sample of nine participants would provide at least 80% power to detect large effect sizes (*f* = 0.5) in the current study (G^*^Power, Düsseldorf, Germany) (35). *Post-hoc*, using the observed data, it was found that a replication would require 10 participants to achieve this power (STATA 16, StataCorp 2019), suggesting that the initial estimate was reasonable. Statistical analyses were performed using Sigma Plot (Version 14.0, Systat Software, Inc., San Jose, CA, USA).

## Results

### Cooling

**Figure 1:**
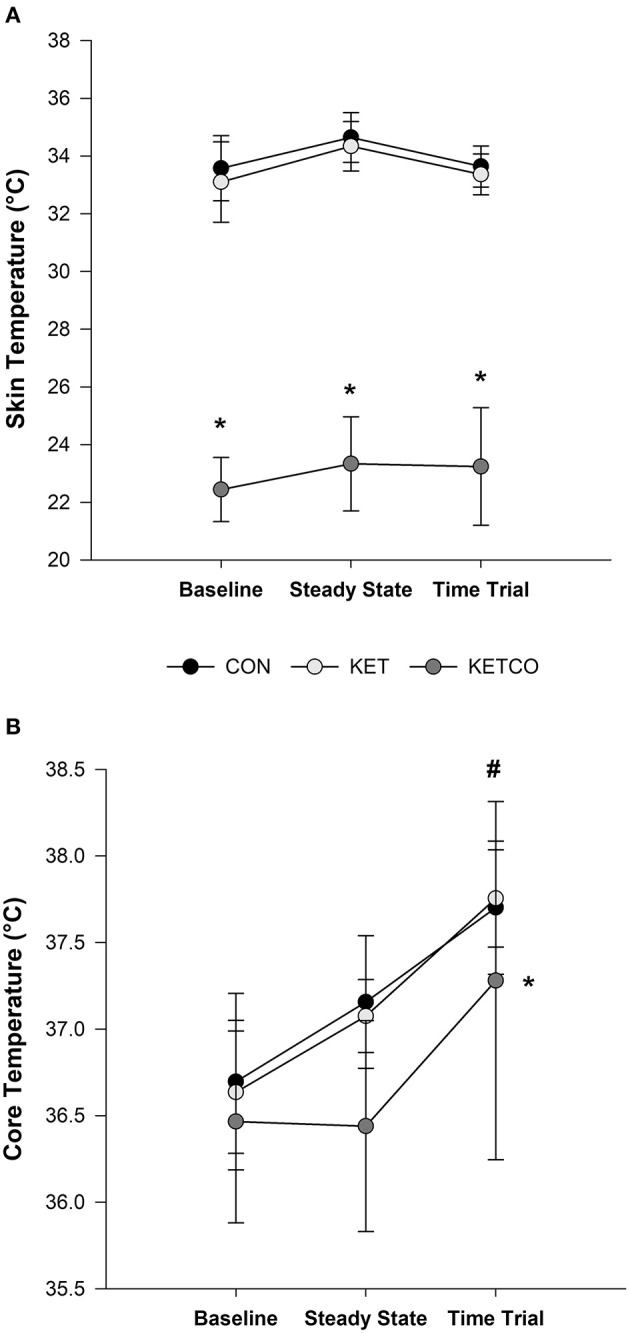
**(A)** Mean skin temperature (${\bar{T}}_{\text{sk}}$), and **(B)** mean core temperature (T_c_) at BL and during SS and TT exercises for each experimental condition. *Significantly different from CON and KET conditions, ^#^significantly different from BL and SS.

A main effect of condition was found for ${\bar{T}}_{\text{sk}}$ (*p* < 0.001) where ${\bar{T}}_{\text{sk}}$ was lower in KETCO (23.3 ± 1.8 °C) compared to both CON (34.1 ± 0.9°C, *p* < 0.001) and KET (33.9 ± 0.9°C, *p* < 0.001). Additionally, there was an interaction between condition and time, where ${\bar{T}}_{\text{sk}}$ was lower in KETCO during both SS and TT exercise (*p* < 0.001) (Figure 1A). A main effect of condition was also observed for T_c_ (*p* = 0.028), which was lower during KETCO (36.7 ± 0.8°C) compared to both CON (37.2 ± 0.6°C, *p* = 0.037) and KET (37.2 ± 0.5°C, *p* = 0.049) (Figure 1B). Further, T_c_ was higher during TT (37.6 ± 0.7°C) compared to BL (36.6 ± 0.5°C, *p* < 0.001) and SS exercise (36.9 ± 0.5°C, *p* = 0.004), regardless of condition.

### Performance and Cardiorespiratory Measures

**Figure 2:**
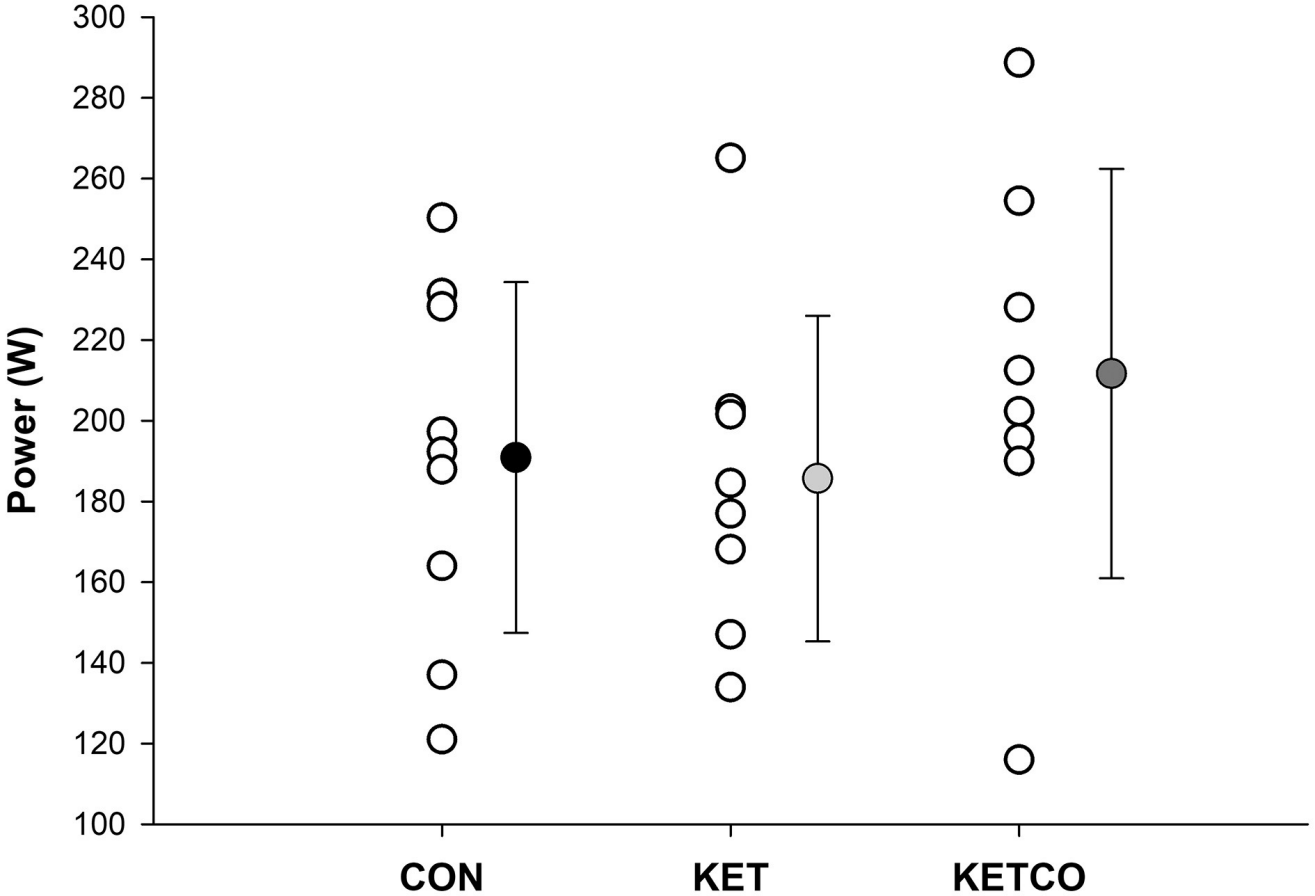
Mean power output during each experimental time trial.

**Figure 3:**
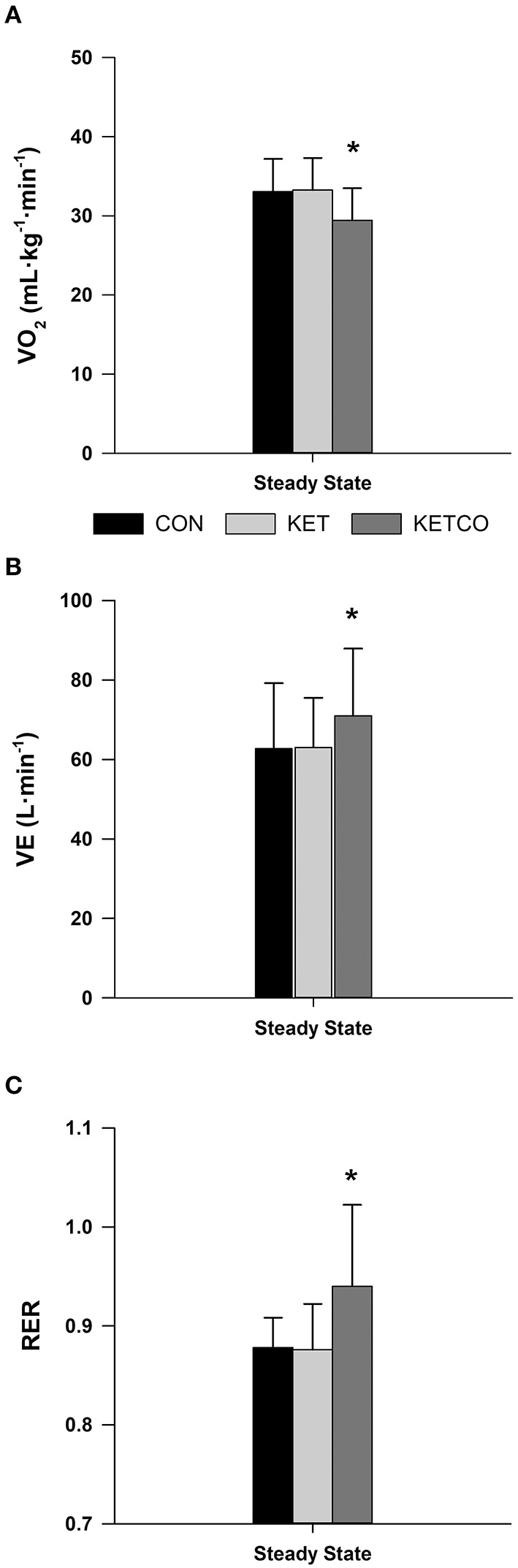
**(A)** Mean oxygen consumption, **(B)** mean minute ventilation, and **(C)** mean respiratory exchange ratio during steady state exercise for each experimental condition. *Significantly different from CON and KET conditions.

Mean power output (Figure 2) during the 15-min time trials was not significantly different between conditions (*p* = 0.112, CON: 190 ± 43.5 W, KET: 185 ± 40.4 W, KETCO: 211 ± 50.7 W). Further, distance traveled during each the TT were not different between conditions (*p* = 0.514, CON: 7,614 ± 383 m, KET: 7,532 ± 503 m, KETCO: 8,067 ± 622 m). The order of the trials did not result in a learning/order effect for power output (*p* = 0.694), or distance traveled (*p* = 0.710) during the TTs. A main effect of condition was observed for mean VO_2_ (*p* = 0.001), which was lower during KETCO (30.6 ± 4.7 ml·min^−1^·kg^−1^) compared to both CON (*p* = 0.007, 35.2 ± 5.2 ml·min^−1^·kg^−1^) and KET (*p* = 0.002, 36.1 ± 5.7 ml·min^−1^·kg^−1^) (Figure 3A). A main effect of condition was overserved for VE (*p* < 0.001), where it was significantly higher in KETCO (83.4 ± 20.3 L·min^−1^) compared to both CON (70.2 ± 15.0 L·min^−1^, *p* < 0.001) and KET (73.4 ± 16.6 L·min^−1^, *p* = 0.005; Figure 3B). Mean RER was also higher in KETCO (main effect of condition, *p* = 0.005) compared to CON (*p* = 0.012) and KET (*p* = 0.014) (Figure 3C).

### Blood Variables

**Figure 4:**
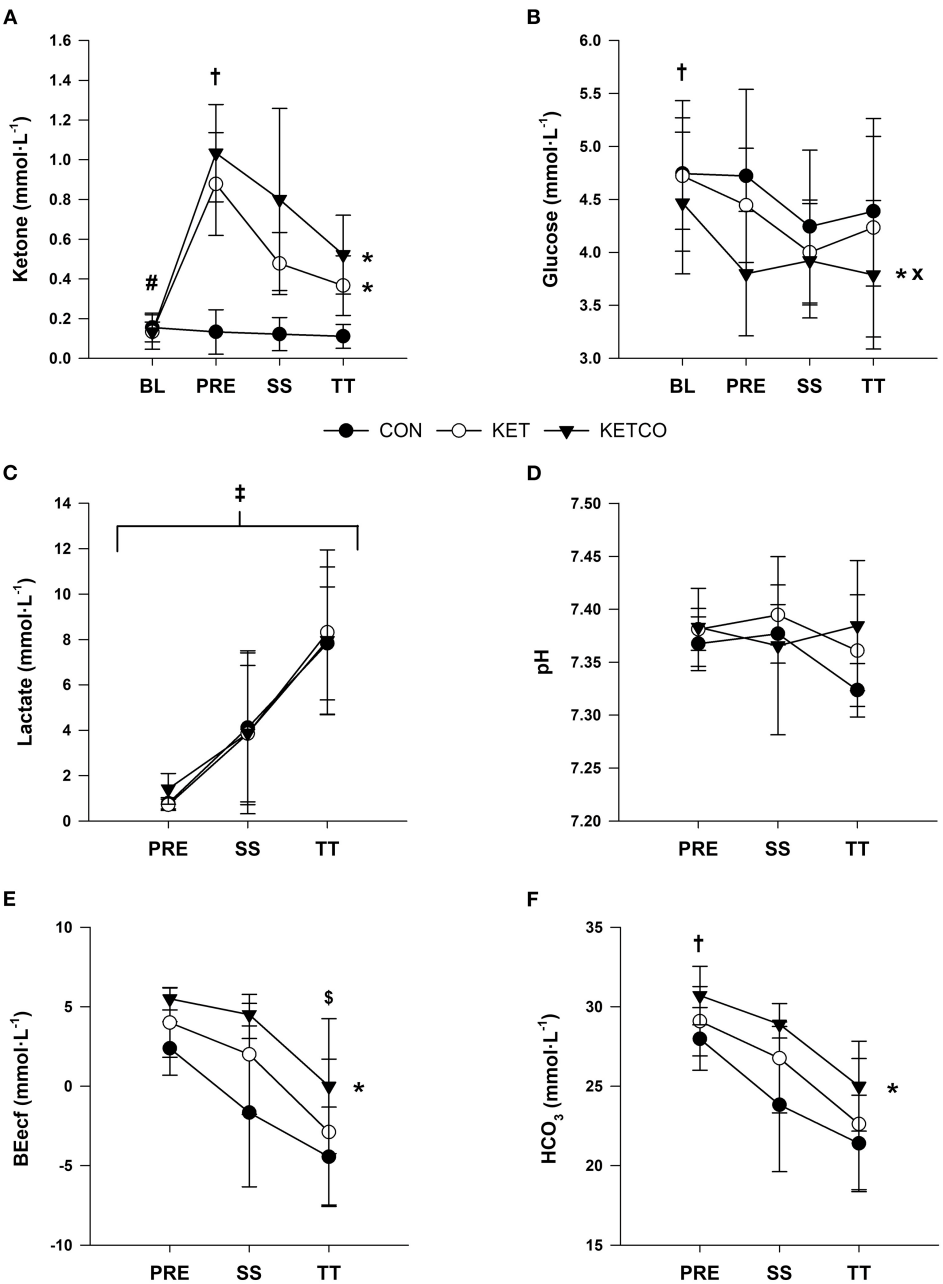
**(A)** Mean ketone (β-OHB) concentrations, **(B)** mean glucose concentrations, **(C)** mean lactate, **(D)** mean pH, **(E)** mean base excess (BEecf), and **(F)** mean bicarbonate (HCO_3_) during each experimental condition at BL, PRE, SS, and TT. *Significantly different from CON, ^#^significantly different from PRE, SS, and TT, significantly different from SS and TT, ^×^significantly different from KET,^‡^significantly different at PRE, SS, and TT, ^$^significantly different from PRE.

At BL, blood β-OHB and glucose concentrations were not different between conditions (*p* ≥ 0.439; Figure 4A). Blood β-OHB were higher at PRE for both KET and KETCO compared to CON (*p* < 0.001). Blood β-OHB concentrations remained elevated at both SS and TT time points for KET and KETCO trials when compared to CON (*p* ≤ 0.004). Blood glucose was significantly lower in KETCO condition compared to both CON (*p* ≤ 0.001) and KET (*p* = 0.01; Figure 4B). Further, blood glucose was higher at BL compared to SS and TT time points (*p* ≤ 0.015), regardless of condition. Blood lactate was not different between conditions (*p* = 0.868, CON: 4.37 ± 3.76 mmol·L^−1^, KET: 4.29 ± 4.12 mmol·L^−1^, KETCO: 4.41 ± 3.85 mmol·L^−1^), but significantly increased at each time point (*p* ≤ 0.013, PRE: 0.98 ± 0.54 mmol·L^−1^, SS: 3.94 ± 1.20 mmol·L^−1^, TT: 8.03 ± 3.04 mmol·L^−1^; Figure 4C). There was no difference in blood pH between conditions (*p* = 0.114) or at any time points (*p* = 0.085; Figure 4D).

BEecf was higher in KETCO (2.3 ± 5.4 mmol·L^−1^) compared to CON (*p* = 0.004, −1.3 ± 4.3 mmol·L^−1^) but not KET (*p* = 0.245, 1.0 ± 4.6 mmol·L^−1^; Figure 4E). Additionally, BEecf was higher at PRE (4.3 ± 5.5 mmol·L^−1^) vs. TT (*p* = 0.002, −3.2 ± 4.1 mmol·L^−1^), but not SS (*p* = 0.108, 0.7 ± 4.8 mmol·L^−1^). HCO_3_ was higher in KETCO (27.2 ± 5.1 mmol·L^−1^) compared to CON (*p* = 0.014, 24.3 ± 4.1 mmol·L^−1^), as well as higher at PRE (29.6 ± 2.5 mmol·L^−1^) compared to SS (*p* = 0.031, 25.4 ± 4.2 mmol·L^−1^) and TT (*p* = 0.001, 22.5 ± 3.7 mmol·L^−1^; Figure 4F).

**Figure 5:**
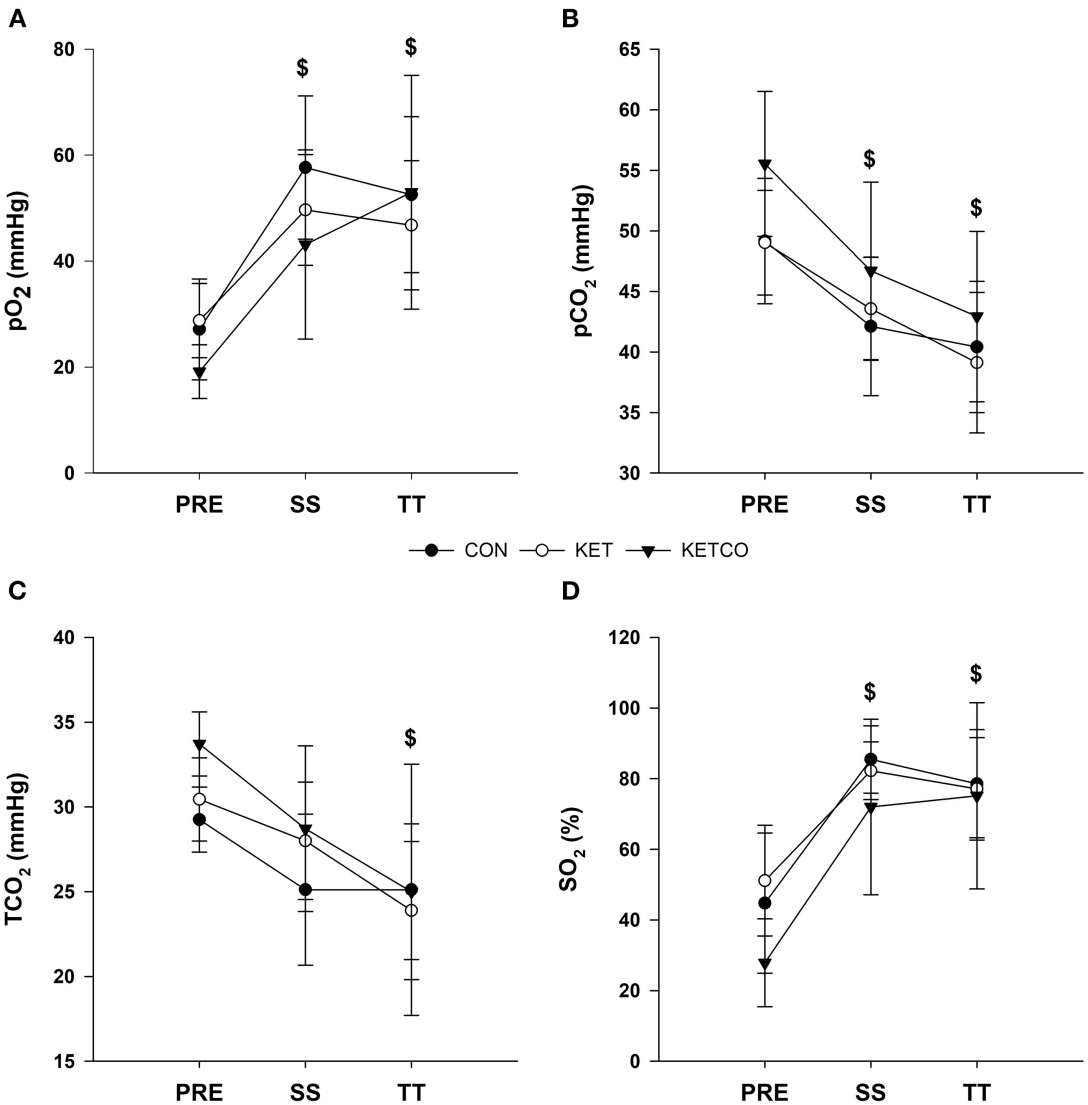
**(A)** Mean venous partial pressure oxygen, **(B)** mean venous partial pressure carbon dioxide, **(C)** mean venous total carbon dioxide concentrations, and **(D)** mean venous oxygen saturation during each experimental condition at PRE, SS, and TT. ^$^Significantly different from PRE.

pO_2_ was lower at PRE (25.4 ± 8.3 mmHg) compared to SS (*p* < 0.001, 50.7 ± 14.6 mmHg) and TT (*p* < 0.001, 50.6 ± 15.9 mmHg) during all conditions (Figure 5A). Likewise, pCO_2_ was higher at PRE (51.0 ± 5.7 mmHg) compared to SS (*p* = 0.007, 43.9 ± 5.8 mmHg) and TT (*p* < 0.001, 40.7 ± 6.0 mmHg) during all conditions (Figure 5B). TCO_2_ was lower at TT (24.6 ± 5.3 mmHg) compared to PRE (*p* = 0.008, 31.0 ± 2.8 mmHg; Figure 5C). SO_2_ was lower at PRE (42.2 ± 18.5 %) compared to both SS (*p* < 0.001, 80.5 ± 15.4 %) and TT (*p* < 0.001, 77.1 ± 18.0 %; Figure 5D).

## Discussion

This study is the first to examine the influence of ketone salt supplementation with whole-body cooling on short-term steady-state aerobic exercise metabolism and time-trial performance. The main findings of the current study were: (1) Ingestion of exogenous ketone salts increased blood ketone concentrations but did not result in apparent changes in metabolism, as indicated by similar RER values during thermoneutral exercise, (2) Physical performance during the time trial was unaltered by ketone salt supplementation alone or when combined with whole-body cooling, and (3) Ketone salt supplementation combined with whole-body cooling increased time trial RER compared to both control and ketone salt supplementation alone.

Mean plasma β-OHB concentrations increased with ketone salt supplementation (KET: 0.88 ± 0.21 mmol·L^−1^, KETCO: 1.03 ± 0.22 mmol·L^−1^) compared to control (CON: 0.13 ± 0.11 mmol·L^−1^), supporting previous work demonstrating that commercially available ketone salts increase blood β-OHB concentrations (13, 15–19). Even though plasma β-OHB was significantly increased, TT performance remained unchanged between conditions, as power output and distance traveled was similar across conditions. Apart from the results from Cox et al. (3), the remainder of the literature on exogenous ketone supplementation (salt and ester) and exercise performance have reported either no benefit (15, 16, 18, 36–41), or negative performance outcomes (13, 42), as identified by recent review and meta-analysis (14). Therefore, the results of this study further demonstrate that supplementation of exogenous ketone salts does not improve short-term aerobic exercise substrate metabolism or high intensity TT performance. However, it is possible that the duration of exercise was not long enough to deplete CHO stores, as blood glucose concentrations were similar between conditions, and therefore CHO availability may not have been a limiting factor. Similarity blood glucose concentrations were matched with no observed differences in blood lactate between conditions. The ability of ketones to lower plasma lactate concentrations during exercise has been demonstrated previously (3, 38, 42), where a decrease in lactate concentrations was observed after supplementation of ketone esters. The attenuation of lactate production *via* exogenous ketosis was proposed as a glucose sparing mechanism (3). However, Poffe et al. (11) recently suggested that reductions in plasma lactate concentrations may result from competition between ketones and lactate for monocarboxylate transporters, limiting the efflux of lactate. It should be noted that the ability for ketones to lower lactate concentrations during exercise has not been shown to occur with ketone salt supplementation (13, 15–19), which may be due to relatively lower blood ketone concentrations limiting the attenuation of glycolysis or transporter competition. Therefore, higher concentrations of blood ketones *via* ketone ester supplementation may be needed to observe this effect. The lack of change in performance during TT exercise was matched with similar mean VO_2_ and RER during KET and CON conditions, indicating that the ketone salts alone also did not produce alterations in metabolism during both SS and TT exercise, however ketone oxidation itself was not directly measured. Inconsistent findings have been observed in other physiological responses to supplementation and exercise, including metabolic and respiratory (14). Thereby, the lack of significant differences between CON and KET in this study adds to the literature that exogenous ketones salts elevate blood ketone concentrations, with no apparent effect on plasma glucose or lactate concentrations, and blood pH, or short duration exercise performance.

Ketone salt supplementation should have theoretically altered substrate oxidation, providing an alternative fuel source to glucose compared to control, particularly because participants were fasted prior to supplementation and exercise. The absence of between-trial differences in RER is consistent with recent ketone salt literature, where participants were fasted (17, 19). It has been suggested that lowering of RER may be masked by the oxidation of ketone bodies, producing RER similar to CHO vs. fats (β-OHB: 0.89). While exogenous ketone salts increase circulating ketones, they also do not reduce CHO availability, which is often observed with endogenous ketosis *via* ketogenic diets or starvation. Therefore, both ketones and CHO substrates may have been readily available for utilization, and it is possible that CHOs may have been the preferred fuel source during exercise (5, 13). Further research should investigate the preference of ketone vs. CHO utilization, following ketone salt supplementation, when both substrates are readily available. Additionally, the ideal skeletal muscle saturation is between 1 and 2 mmol·L^−1^ (43). However, maximum ketone clearance has been shown to occur at blood ketone concentrations of ~2 mmol·L^−1^ (43) and when ketone concentrations are raised from 2 to 4 mmol·L^−1^, rate of ketone oxidation is unchanged (44). Therefore, ketone salt supplementation may not have increased plasma concentrations enough to result in optimal ketone uptake utilization by muscle tissue during exercise. The two key ketolytic enzymes responsible for the utilization of ketone are catalyst 3-hydroxybutryate dehydrogenase (BDH), which reduces β-OHB back into acetoacetate, and succinyl-CoA: 3-oxoacid CoA transferase (OXCT), which transfers acetoacetate into Acetyl Co-A to be used in the Krebs cycle (5, 10). These two enzymes are higher in trained muscle, as well as in type I, slow twitch muscle fibers (45), and it has recently been demonstrated that percentage of type I muscle fibers had a positive associate with rate of ketones oxidation (44). Therefore, individuals who have a predominance of type I, slow twitch fibers, or well-trained athletes, might be more suitable to gain benefits from ketones, rather than the recreational athletes who participated in the current study.

Interestingly, when ketone salts were combined with cold exposure (KETCO), VO_2_ was lowered, and an increase in RER was observed compared to ketone salt supplementation alone (KET). Cold exposure during exercise has been shown to increase whole-body lipolysis and lipid oxidation (7, 8). However, when shivering thermogenesis is initiated, skeletal muscles rely primarily on CHO metabolism, significantly increasing CHO oxidation by as much as 558% (46). In the current study, once exercise was initiated, cooling continued *via* an ice vest worn by the participant. Non-active muscles likely stayed in a thermogenic state during exercise, continuing to consume CHO in order to produce the necessary body heat, as indicated by the high RER in the KETCO condition (1.003 ± 0.091) vs. KET (0.885 ± 0.052). Febbraio et al. (6) observed a higher RER during submaximal exercise in the cold (3°C) vs. room temperature (20°C), even though localized decreases in CHO metabolism were observed within the active muscles (vastus lateralis). Even though we did not measure insulin, shivering thermogenesis may also be responsible for lower circulating glucose concentrations during KETCO vs. KET, both after supplementation (PRE) and at the end of the time trial (TT). The initiation and continuation of shivering thermogenesis, due to precooling and percooling, respectively, may have overshadowed any localized increase in ketone or fat metabolism within the active muscles, due to an even larger increase in total body CHO metabolism, resulting in an elevated RER during KETCO.

We also observed an increase in V_E_ in KETCO compared to KET. An upward shift in the V_E_/VO_2_ curve is generally influenced by pulmonary hypertension, or rather, a higher pulmonary arterial tension from lower circulatory capacity (47, 48). Our protocol used a cold environment and a cold ice-packed vest for whole-body cooling, with the presumption that the use of a cooling vest has shown increases in human performance (49). Since core cooling was significant across SS and TT, it is probable that direct cooling of the torso, while remaining in the cold, could have reduced pulmonary blood flow, and lower ventilatory efficiency.

The methods used to measure blood ketone concentrations in this study could only evaluate concentrations of R-β-OHB, and therefore do not reflect the total concentration of ketones during the trials. Even though experimental conditions were completed in a balanced design, the participants in this study did not complete a familiarization trial of the experimental protocol. The lack of a familiarization may have increased the variation in the time trial data. It is possible that cooling on its own may have negatively impacted performance through changes in respiratory and muscle functions, however, this study did not include a cooling only condition and therefore we cannot draw direct conclusions to the effects of cooling on performance in the absence of ketone salt supplementation. Additionally, the thermoregulatory results of this study may not be generalizable to precooling prior to performance in hot and humid environments, as the ambient temperature during exercise was ~21°C. Men and women present clear metabolic differences (50). The present study contained information from males only, preventing us from generalizing the current data set to women. Additionally, even though the participants in this study were aerobically fit, they were not trained endurance athletes, and adaptations associated with endurance training may result in different metabolic and performance outcomes. The power analysis revealed that a sample size of 9 would be sufficient, however a larger sample size may have revealed differences between conditions. Moreover, while we ensured participants consumed the same diet the day before each experimental visit, we did not collect these records. Accordingly, blood analyses revealed that participants were not following a ketogenic diet, but we cannot determine if participants were consuming high-fat or high-CHO diets, or the potential impact of diet on our results. Finally, the lack of skeletal muscle biochemical analyses to assess glycolytic, lipolytic, and ketogenic fluxes during the exercise protocol makes it challenging to offer definitive conclusions to the present results.

## Conclusions

The present study demonstrates that ketone salts supplementation does not lead to improvements in TT performance. Furthermore, ketone salt supplementation alone did not alter plasma glucose availability or lactate concentrations, suggesting that modest increases in circulating ketones (0.88 ± 0.21 mmol·L^−1^) from ketone salt supplementation in healthy, non-endurance trained individuals, are insufficient to provide a substantial shift in energy metabolism during exercise. Finally, the combination of ketone salt supplementation with precooling and percooling increased circulating ketones (1.03 ± 0.22 mmol·L^−1^) but did not lead to an increase in performance compared to ketone salts alone.

## Data Availability Statement

The raw data supporting the conclusions of this article will be made available by the authors, without undue reservation.

## Ethics Statement

The studies involving human participants were reviewed and approved by Laurentian University Ethics Review Board. The patients/participants provided their written informed consent to participate in this study.

## Author Contributions

DC and DG: conceptualization, methodology, and data collection. DC and SM: formal analysis and writing—original draft preparation. DC, SM, K-HH, and DG: writing—review and editing. SM and K-HH: visualization. KH and DG: supervision. DG: funding acquisition. All authors have read and agreed to the published version of the manuscript.

## Conflict of Interest

The authors declare that the research was conducted in the absence of any commercial or financial relationships that could be construed as a potential conflict of interest.

## References

[1] Bongers CCWG Thijssen DHJ Veltmeijer MTW Hopman MTE Eijsvogels TMH. Precooling and percooling (cooling during exercise) both improve performance in the heat: a meta-analytical review. Br J Sports Med. (2015) 49:377–84. 10.1136/bjsports-2013-092928 24747298

[2] Fraczek B Warzecha M Tyrala F Pieta A. Prevalence of the use of effective ergogenic aids among professional athletes. Rocz Państw Zakl Hig. (2016) 67:271–8. 27546324

[3] Cox PJ Kirk T Ashmore T Willerton K Evans R Smith A. Nutritional ketosis alters fuel preference and thereby endurance performance in athletes. Cell Metab. (2016) 24:256–68. 10.1016/j.cmet.2016.07.010 27475046

[4] Egan B D'Agostino DP. Fueling performance: ketones enter the mix. Cell Metab. (2016) 24:373–5. 10.1016/j.cmet.2016.08.021 27626197

[5] Evans M Cogan KE Egan B. Metabolism of ketone bodies during exercise and training: Physiological basis for exogenous supplementation. J Physiol. (2017) 595:2857–71. 10.1113/JP273185 27861911 PMC5407977

[6] Febbraio MA Snow RJ Stathis CG Hargreaves M Carey MF. Blunting the rise in body temperature reduces muscle glycogenolysis during exercise in humans. Exp Physiol. (1996) 81:685–93. 10.1113/expphysiol.1996.sp003969 8853276

[7] Gagnon DD Rintamäki H Gagnon SS Cheung SS Herzig KH Porvari K. Cold exposure enhances fat utilization but not non-esterified fatty acids, glycerol or catecholamines availability during submaximal walking and running. Front Physiol. (2013) 4:99. 10.3389/fphys.2013.00099 23675353 PMC3650516

[8] Gagnon DD Perrier L Dorman SC Oddson B Larivière C Serresse O. Ambient temperature influences metabolic substrate oxidation curves during running and cycling in healthy men. Eur J Sport Sci. (2019) 20:90–9. 10.1080/17461391.2019.1612949 31079551

[9] Sato K Kashiwaya Y Keon CA Tsuchiya N King MT Radda B. Insulin, ketone bodies, and mitochondrial energy transduction. FASEB J. (1995) 9:651–8. 10.1096/fasebj.9.8.7768357 7768357

[10] Cox PJ Clarke K. Acute nutritional ketosis: implications for exercise performance and metabolism. Extreme Physiol Med. (2014) 3:17. 10.1186/2046-7648-3-17 25379174 PMC4212585

[11] Poffe C Ramaekers M Bogaerts S Hespel P. Exogenous ketosis impacts neither performance nor muscle glycogen breakdown in prolonged endurance exercise. J Appl Physiol. (2020) 128:1643–53. 10.1152/japplphysiol.00092.2020 32407242 PMC7311686

[12] Kesl SL Poff AM Ward NP Fiorelli TN Ari C Van Putten AJ. Effects of exogenous ketone supplementation on blood ketone, glucose, triglyceride, and lipoprotein levels in Sprague-Dawley rats. Nutr Metab. (2016) 13:9. 10.1186/s12986-016-0069-y 26855664 PMC4743170

[13] O'Malley T Myette-Cote E Durrer C Little JP. Nutritional ketone salts increase fat oxidation but impair high-intensity exercise performance in healthy adult males. Appl Physiol Nutr Metab. (2017) 42:1031–5. 10.1139/apnm-2016-0641 28750585

[14] Valenzuela PL Morales JS Castillo-Garcia A Lucia A. Ketone supplementation and exercise performance: a systematic review and meta-analysis of randomized controlled trials. Int J Sports Physiol Perform. (2020) 15:298–308. 10.1123/ijspp.2019-0918 32045881

[15] Rodger S Plews D Laursen P Driller MW. Oral β-hydroxybutyrate salt fails to improve 4-minute cycling performance following submaximal exercise. J Sci Cycling. (2017) 6:26–31.

[16] Waldman HS Basham SA Price FG Smith JEW Chander H Knight AC. Exogenous ketone salts do not improve cognitive responses after a high-intensity exercise protocol in healthy college-aged males. Appl Physiol Nutr Metab. (2018) 43:711–7. 10.1139/apnm-2017-0724 29451991

[17] Prins PJ D'Agostino DP Rogers CQ Ault DL Welton GL Jones DW. Dose response of a novel exogenous ketone supplement on physiological, perceptual and performance parameters. Nutr Metab (Lond). (2020) 17:81. 10.1186/s12986-020-00497-1 33005207 PMC7523040

[18] Evans M Patchett E Nally R Kearns R Larney M Egan B. Effect of acute ingestion of β-hydroxybutyrate salts on the response to graded exercise in trained cyclists. Eur J Sport Sci. (2018) 18:376–86. 10.1080/17461391.2017.1421711 29338584

[19] Prins PJ Koutnik AP D'Agostino DP Rogers CQ Seibert JF Breckenridge JA. Effects of an exogenous ketone supplement on five-kilometer running performance. J Hum Kinet. (2020) 72:115–27. 10.2478/hukin-2019-0114 32269653 PMC7126257

[20] Galloway SD Maughan RJ. Effects of ambient temperature on the capacity to perform prolonged cycle exercise in man. Med Sci Sports Exerc. (1997) 29:1240–9. 10.1097/00005768-199709000-00018 9309637

[21] Peiffer JJ Abbiss CR. Influence of environmental temperature on 40 km cycling time-trial performance. Int J Sports Physiol Perform. (2011) 6:208–20. 10.1123/ijspp.6.2.208 21725106

[22] Febbraio MA Snow RJ Stathis CG Hargreaves M Carey MF. Effect of heat stress on muscle energy metabolism during exercise. J Appl Physiol. (1994) 77:2827–31. 10.1152/jappl.1994.77.6.2827 7896628

[23] Hargreaves M Angus D Howlett K Conus NM Febbraio M. Effect of heat stress on glucose kinetics during exercise. J Appl Physiol. (1996) 81:1594–7. 10.1152/jappl.1996.81.4.1594 8904574

[24] Hessemer V Langusch D Brück K Bödeker RH Breidenbach T. Effect of slightly lowered body temperatures on endurance performance in humans. J Appl Physiol. (1984) 57:1731–7. 10.1152/jappl.1984.57.6.1731 6096319

[25] Lee DT Haymes EM. Exercise duration and thermoregulatory responses after whole bod precooling. J Appl Physiol. (1995) 79:1971–6. 10.1152/jappl.1995.79.6.1971 8847262

[26] Olschewski H Bruck K. Thermoregulatory, cardiovascular, and muscular factors related to exercise after precooling. J Appl Physiol. (1988) 64:803–11. 10.1152/jappl.1988.64.2.803 3372438

[27] Marsh D Sleivert G. Effect of precooling on high intensity cycling performance. Br J Sports Med. (1999) 33:393–7. 10.1136/bjsm.33.6.393 10597847 PMC1756209

[28] Shepard RJ. Metabolic adaptations to exercise in the cold. Sports Med. (1993) 16:266–89. 10.2165/00007256-199316040-00005 8248684

[29] Castellani JW Young AJ Sawka MN Pandolf KB. Human thermoregulatory responses during serial cold-water immersions. J Appl Physiol. (1998) 85:204–9. 10.1152/jappl.1998.85.1.204 9655776

[30] Jeacocke NA Burke LM. Methods to standardize dietary intake before performance testing. Int J Sport Nutr Exerc Metab. (2010) 20:87–103. 10.1123/ijsnem.20.2.87 20479482

[31] Bailey CP Hennessy E. A review of the ketogenic diet for endurance athletes: performance enhancer or placebo effect? J Int Soc Sports Nutr. (2020) 17:33. 10.1186/s12970-020-00362-9 32571422 PMC7310409

[32] Jackson AS Pollock ML. Generalized equations for predicting body density. Br J Nutr. (1978) 40:497–504. 10.1079/BJN19780152 718832

[33] Stubbs BJ Cox PJ Evans RD Santer P Miller JJ Faull OK. On the metabolism of exogenous ketones in humans. Front Physiol. (2017) 8:1–13. 10.3389/fphys.2017.00848 29163194 PMC5670148

[34] Ramanathan NL. A new weighting system for mean surface temperature of the human body. J Appl Physiol. (1964) 19:531–3. 10.1152/jappl.1964.19.3.531 14173555

[35] Jeukendrup AE Wallis GA. Measurement of substrate oxidation during exercise by means of gas exchange measurements. Int J Sports Med. (2004) 26:S28–S37. 10.1055/s-2004-830512 15702454

[36] Scott BE Laursen PB James LJ Boxer B Chandler Z Lam E. The effect of 1,3-butanediol and carbohydrate supplementation on running performance. J Sci Med Sport. (2019) 22:702–6. 10.1016/j.jsams.2018.11.027 30553764

[37] Dearlove DJ Faull OK Rolls E Clarke K Cox PJ. Nutritional ketoacidosis during incremental exercise in healthy athletes. Front Physiol. (2019) 10:290. 10.3389/fphys.2019.00290 30984015 PMC6450328

[38] Evans M Egan B. Intermittent running and cognitive performance after ketone ester ingestion. Med Sci Sports Exerc. (2018) 50:2330–8. 10.1249/MSS.0000000000001700 29944604

[39] Evans M McSwiney FT Brady AJ Egan B. No benefit of ingestion of a Ketone monoester supplement on 10-km running performance. Med Sci Sports Exerc. (2019) 51:2506–15. 10.1249/MSS.0000000000002065 31730565

[40] Faull OK Dearlove DJ Clarke K Cox PJ. Beyond RPE: the perception of exercise under normal and ketotic conditions. Front Physiol. (2019) 10:229. 10.3389/fphys.2019.00229 30941052 PMC6433983

[41] Shaw D Merien F Braakhuis A Plews D Laursen P Dulson D. The effect of 1, 3-butanediol on cycling time-trial performance. Int J Sport Nutr Exerc Metab. (2019) 29:466–73. 10.1123/ijsnem.2018-0284 30632425

[42] Leckey JJ Ross ML Quod M Hawley JA Burke LM. Ketone diester ingestion impairs time-trial performance in professional cyclists. Front Physiol. (2017) 8:1–10. 10.3389/fphys.2017.00806 29109686 PMC5660098

[43] Balasse EO Fery F. Ketone body production and disposal: effects of fasting, diabetes, and exercise. Diabetes Metab Rev. (1989) 5:247–70. 10.1002/dmr.5610050304 2656155

[44] Dearlove DJ Harrison OK Hodson L Jefferson A Clarke K Cox PJ. The effect of blood ketone concentration and exercise intensity on exogenous ketone oxidation rates in athletes. Med Sci Sports Exerc. (2021) 53:505–16. 10.1249/MSS.0000000000002502 32868580 PMC7886359

[45] Winder WW Baldwin KM Holloszy JO. Enzymes involved in ketone utilization in different types of muscle: adaptations to exercise. Eur J Biochem. (1974) 47:461–7. 10.1111/j.1432-1033.1974.tb03713.x 4373244

[46] Vallerand AL Jacobs I. Rates of energy substrates utilization during human cold exposure. Eur J Appl Physiol Occup Physiol. (1989) 58:873–8. 10.1007/BF02332221 2767069

[47] Murphy RM Weiner RB Hough SS Pappagianopoulos PP Systrom D Hutter A. Determinants of the VE/VCO2 slope in normal individuals; Ventilatory efficiency is modifiable with endurance training. J Am Coll Cardiol. (2019) 59:E1943. 10.1016/S0735-1097(12)61944-5

[48] Riley MS Porszasz J Engelen MPKJ Brundage BH Wasserman K. Gas exchange responses to continuous incremental cycle ergometry exercise in primary pulmonary hypertension in humans. Eur J Applied Physiol. (2000) 83:63–70. 10.1007/s004210000240 11072775

[49] Duffield R Dawson B Bishop D Fitzsimons M Lawrence S. Effect of wearing an ice cooling jacket on repeat sprint performance in warm/humid conditions. Br J Sports Med. (2003) 37:164–9. 10.1136/bjsm.37.2.164 12663361 PMC1724622

[50] Venables MC Achten J Jeukendrup AE. Determinants of fat oxidation during exercise in healthy men and women: a cross-sectional study. J Appl Physiol. (2005) 98:160–7. 10.1152/japplphysiol.00662.2003 15333616

